# Predicting determinants of modern contraceptive use among reproductive-age women in Ethiopia using machine learning algorithms: Evidence from the Performance Monitoring and Accountability (PMA) Survey 2019 dataset

**DOI:** 10.12688/f1000research.156316.2

**Published:** 2025-11-28

**Authors:** Jibril Bashir Adem, Anas Ali Alhur, Shimels Derso Kebede, Agmasie Damtew Walle, Daniel Niguse Mamo

**Affiliations:** 1 Public Health, Arsi University, Asella, Oromia, 193, Ethiopia; 2 Department of Health Information Management and Technology, College of Public Health, Imam Abdulrahman Bin Faisal University, Dammam, Saudi Arabia; 3 health Informatics, Wollo University, Dessie, Amhara, Ethiopia; 4 Health Informatics, Debre Berhan University, Debre Birhan, Amhara, Ethiopia; 5 Health Informatics, Arba Minch University, Arba Minch, Southern Nations, Nationalities, and People's Region, Ethiopia

**Keywords:** Modern contraceptive methods, Reproductive-age women, Machine learning approach, Ethiopia

## Abstract

**Introduction:**

Globally, around 40% of women report unintended pregnancies, with approximately 214 million women in developing countries wanting to avoid pregnancy but not using any contraception. Modern contraceptives (MCs) are effective tools for preventing unintended pregnancies, controlling rapid population growth, and reducing fertility and maternal mortality rates, particularly in developing countries. This study aimed to identify the determinants of modern contraceptive use among Ethiopian women of reproductive age using machine learning (ML) algorithms.

**Methodology:**

The study utilized secondary data from the 2019 Performance Monitoring and Accountability (PMA) Ethiopia survey, analyzing 8,837 samples. Preprocessing steps included data cleaning, feature engineering, dimensionality reduction, and splitting the data, with 80% used for training and 20% for testing the algorithms. Six supervised ML algorithms were employed and assessed using confusion matrices, with information gain applied to identify critical attributes for predicting MC use.

**Results:**

Only 24% of participants used modern contraceptives [95% CI (23.1%, 24.9%)]. Extreme gradient boosting (XGB) demonstrated the highest predictive accuracy (81.97%, 95% CI {79.06%, 82.7%}) and area under the ROC curve (76.63%), followed by logistic regression (80.52%) and support vector machines (80.41%). Key determinants of MC use included starting family planning at age 20 or older, being single, having partner approval, being the wife of the household head, being between 36 and 49 years old, advice from healthcare providers, concerns about side effects, and having a household size of five or more.

**Conclusion and Recommendations:**

The use of modern contraceptives among Ethiopian women remains low. Extreme gradient boosting proved most effective in predicting determinants of MC use. Based on the results of predictive associations, improved counseling during antenatal and postnatal care visits, promoting partner discussions on family planning, and addressing concerns about family size and contraceptive use are recommended strategies to enhance MC uptake.

**Abbreviations**
ANC: Antenatal Care
AUC: Area under the ROC curve
DHS: Demographic and Health Survey
EDHS: Ethiopian Demographic and Health Survey
EPHI: Ethiopian Public Health Institute
FP: Family Planning
KNN: K-nearest neighbors
LR: Logistic Regression
NB: Naive Bayes
PMA: Performance monitoring and accountability
PNC: Postnatal care
RF: Random Forest

## Introduction

Approximately 40% of women worldwide report having unwanted pregnancies. ^1^ In developing nations, an estimated 214 million women of reproductive age who want to avoid pregnancy do not use any method of contraception. ^2^ It is estimated that contraception prevents about 188 million unintended pregnancies each year, and in 2022 alone, contraception prevented more than 141 million unintended pregnancies globally. ^3, 4^ Although this expectation has not yet been met in Sub-Saharan Africa, it has been observed in many other regions of the world, particularly in Asia and Latin America. ^5^ Sub-Saharan Africa as a whole still has the highest fertility rate worldwide. ^5^ According to reports from 2012 and 2017, only a small percentage of women in Africa used modern contraceptives, with estimates of 23.9% and 28.5%, respectively. ^6^ In a recent large population-based study, the prevalence of modern contraceptive use was estimated to be 26% among women of reproductive age in 20 African countries, with country-specific variations ranging from 6% in Guinea to 62% in Zimbabwe. ^7^

Modern contraceptive (MC) methods are a scientifically effective methods like implants, female and male condoms, injectable, contraceptive pills, standard days method, male and female sterilization, intrauterine devices, and emergency contraception to control the fertility of reproductive-aged groups of people. ^8– 10^ MCs are widely accepted to limit rapid population increases, especially in developing nations, and have been proven to be an effective approach for reducing fertility. ^11, 12^ Effective use of MCs has been shown to improve birth spacing, reduce the rate of unexpected or unwanted pregnancies, lower unsafe abortions, improve maternal health, reduce infant mortality, and prevent STDs. ^13, 14^ The decreases in poverty, the expansion of women’s educational possibilities, and the ensuing sustainable population growth and economic development for nations are among the non-health benefits that have been identified. ^15^

According to a study conducted in Vietnam, the association between a woman’s age and current contraceptive methods is shaped like an inverted “U.” Although the chance of using contraception was low among women aged 15 to 24, it was greater among those in the 25 to 35 age group and lowest among those aged 35 and older. ^16^ The adoption and use of modern contraceptive methods by women can also be influenced by their degree of education. Employed women with higher educational levels had a noticeably greater likelihood of using contraceptives than illiterate women did, according to a study of the prevalence and determinants of contraceptive usage among employed and jobless women. ^17^ According to a Nigerian study, women with higher (tertiary) education are four times more likely to use modern contraceptives than women with lower educational attainment. ^18^ In a similar vein, wives of highly educated men were more likely to accept and support the use of modern contraceptive techniques. ^19^

Numerous investigations have shown a strong correlation between place of residence and the use of modern contraceptive methods. ^20, 21^ Women in urban regions are more likely than women in rural areas to use modern contraceptive techniques, although the majority of people live in rural areas. ^22^ In relation to modern contraceptives, a woman’s wealth index and type of wage impact her financial status as well as her accessibility and affordability. ^23^ The acceptance and use of a modern contraceptive by a woman can be influenced by her marital status. ^24^ The choice and use of modern contraceptive methods have been linked to cultural influences, religion, and information sources, all of which have an impact on women’s decisions. ^25^

To pinpoint the causes of the low use of modern contraceptives, numerous studies have been conducted in Ethiopia and other countries. ^20, 21^ Their findings suggest that low use of contraceptives is responsible for the high fertility rates in sub-Saharan African nations, which have an adverse impact on early childbirth, high infant and maternal mortality, and a host of other socioeconomic factors. ^26, 27^ Using traditional regression models, earlier research conducted in this country demonstrated the effects of socioeconomic and demographic factors related to the use of modern contraceptives, which became less accurate as the number of variables used and the potential correlations increased. ^28, 29^ These traditional models usually involve problems involving multidisciplinary relationships between variables and many factors. ^30, 31^

This study employed machine learning (ML) to address the limitations of traditional regression models, such as logistic regression, which assume linear relationships and independence among predictors, assumptions that may not be valid in complex public health datasets. In contrast, ML algorithms can efficiently handle nonlinear associations, multicolinearity, and higher-order interaction effects between socio-demographic, behavioral, and reproductive factors influencing contraceptive use. These methods thus provide greater flexibility in uncovering hidden patterns and improving predictive accuracy compared to conventional models. ^30, 32, 33^ Therefore, this study aimed to assess the determinants of modern contraceptive use among reproductive-aged women in Ethiopia using six widely used machine learning (ML) algorithms. This study sought to determine and identify consistent determinants and others of modern contraceptive use using the Performance Monitoring and Accountability (PMA) Survey 2019 dataset for currently non-pregnant reproductive-age women in Ethiopia. The most influential and consistent determinants identified based on these findings will serve as priority intervention areas for which the Ethiopian Ministry of Health and other health partners can concentrate to improve the use of modern contraceptives in Ethiopia.

## Method

### Study design

A machine learning (ML) algorithm was conducted using Python and analyzed on Google Colab, ^34, 35^ utilizing secondary data from the 2019 Performance Monitoring and Accountability (PMA) Ethiopia cross-sectional household and women’s survey. PMA-Ethiopia is a collaborative five-year initiative (2019–2023) involving Addis Ababa University, Johns Hopkins University, and the Federal Ministry of Health. The project comprises three key components: annual cross-sectional surveys of women aged 15–49, longitudinal studies tracking pregnant women and new mothers, and yearly service delivery point surveys assessing health facilities. ^36^

### Source and study population

The study sourced all reproductive-age women in Ethiopia, with the study population comprising women who participated in the 2019 PMA-Ethiopia cross-sectional survey.

### Sampling method

The PMA survey sample is based on a multi-stage cluster design, with urban-rural and primary fields as strata. A nationally representative number of enumeration areas are selected from each region of the country. Then, in each enumeration region, households are identified and mapped before being systematically chosen for participation in the survey round through a random process. The female participant survey is included in each household questionnaire and consists of a series of questions for all women of reproductive age (15-49) residing in that household.

The household and female surveys are carried out by female data collectors, known as resident enumerators (REs) which are typically women over the age of 21 who are from or near the respective enumeration areas and hold at least a high school diploma. Each RE takes about six weeks to collect data from all selected households, eligible women, and service delivery points. The surveys include interviews among females aged 15 to 49 who are consented and screened for eligibility, as well as a random sample of health institutions, pharmacies, and retail stores that provide family planning services in the selected areas.

Women are eligible for the survey if they are regular members of the household, including women staying at their parental home for the delivery and postpartum period, and self-identified as currently pregnant or less than six weeks postpartum. Female respondents are asked questions about their background, birth history, fertility desires, methods of contraception used, and other information that policymakers and program administrators may utilize to promote health and family planning. ^36^ The analysis sample was weighted to account for nonresponse and differences in selection probabilities. It was further limited to responses from women of reproductive age at the time of the survey, resulting in a weighted sample of 8,837 women.

### Study variables

**Dependent variable**

In this study, Modern contraceptive use was the dependent variable and was dichotomized into two categories: ‘yes’ and ‘No’. A woman was considered to be using modern contraception if she used any of the following methods; female sterilization, implant, IUD, injectable, pill, emergency contraception, female or male condoms, cycle beads, and LAM. ^37^

**Predictor variables**

Various socio-demographic, economic, maternal and health service-related factors were included as predictor variables. Socio-demographic and economic factors included mothers’ current age (categorized in to 15-24 years, 25-34 years and 35-49 years), region (included Gambela, Harari, Southern Nations Nationalities and People (SNNP), Oromia, Somalia, Benishangul-Gumuz, Afar, Amhara, Tigray, and Afar and two cities namely Addis Ababa and Dire Dawa), residence (urban and rural), religion (Christian, Muslim and Other which includes Wakefata and Atheist) and educational level (categorized as no schooling, primary education, and secondary and above), sex of the household head (Male and Female), and relationship (grouped in to head of the household, wife, daughter or daughter-in-law, other relatives and not related), family wealth indices (poorest, middle and richest), household size( grouped in to 1-3 members, 4-5 members, 6 and above members) and marital status(grouped in to never married, Married and widowed/divorced/separated).

Maternal and health service-related factors included number of under five children (grouped in to no under_5 children and 1 and more under_5 children), number of children ever born (grouped in to 1-3 children and 4 and more children), birth in the last 5 years (1-2 birth and 3-5 birth), birth in the last year (no birth and 1 birth), knowledge about reproductive methods (knows about modern methods, knows about traditional methods only, and no knowledge about any method), cesarean delivery (no and yes) and age at first birth (below 15 years, between 16 and 34 years and above 35 years) ( Table 1).

**Table 1.** Variables and their descriptions.

Number	Features	Features’ description
1.	Region	Region of the women, All administrative region in Ethiopia
2.	Religion	Religion of the women
3.	Education level	Highest educational level of the women during data collection
4.	wealth index	Wealth index of the HH
5.	Media access	Media access of the women
6.	Ever been pregnant	Women’s history of pregnancy
7.	Age at first sex	Age at sexual initiation
8.	Ever used FP methods	History of FP use
9.	Ever delivered in health facility	History of facility delivery
10.	know any contraceptive method available	Knowledge of contraceptive method
11.	Ageat1stfpuse	Age at first family planning use
12.	Partner_fp_feeling	Partner feeling toward FP
13.	Visited _ a_ facility	Previous health facility visit history
14.	Relationship	Women’s relationship to head of the household
15.	Wge _ fp _ aut_ could_ conflict	If I use FP, there could be conflict in relationship or marriage
16.	facility _ fp _ discussion	health care providers spoke about FP methods at health facility visits
17.	newage	Recoded age
18.	Norm _fp_ responsible	perceptions of couples using FPs as financially responsible
19.	Partner _ discussion	Culture of family planning discussion with partner
20.	Wge _ fp _ aut_ sideeffect _ disrupt	If I use FP, side effects might disrupt my relationship

### Data processing and analysis

Variable extraction and imputation were conducted using R software version 4.4.3. Using Jupyter Notebook, the Scikit-learn machine learning (ML) algorithms were built in Python 3.11.5. We used six widely accepted machine learning algorithms to predict the determinants of modern contraceptive use in Ethiopia and compared the results of the best algorithm to the results of the traditional logistic regression model. The k-nearest neighbors (KNN) model is chosen based on its ability to detect linear and nonlinear boundaries between groups. The K value represents the number of nearest neighbors and is the core deciding factor in this classifier. The Random Forest (RF) model is used in machine learning situations because it is highly flexible and provides good predictive performance. It produces ensemble predictions that are more accurate than any of the individual predictions. The naive Bayes algorithm is a supervised machine learning algorithm that uses the Bayes theorem for classification and prediction. It has an incremental learning behavior and is not affected by training time. Logistic regression is a statistical model that is used to classify and predict different health parameters, where the target variable is dichotomous and the independent variables are independent of each other. The general framework used in the literature ^38, 39^ based on Yufeng Guo’s 7 machine learning steps was used in this study. The framework describes the seven steps in supervised machine learning, which are as follows: data collection, data preparation, model selection, model training, model evaluation, parameter tuning, and prediction.

**Data source/collection**

The dataset for this study is available on the PMA Survey website and can be obtained upon formal request. A weighted sample of 8837 reproductive-age women was included in the data. The datasets analyzed in the current study are available in the PMA repository, https://www.pmadata.org/data/available-datasets .

**Data preparation:** The data preprocessing techniques used in this investigation included data cleaning, data splitting, feature engineering, and dimensionality reduction. After the data were extracted, data cleaning was performed, which included finding and removing outliers from the dataset, as well as resolving missing values and uneven categories in the resulting variable. This study employed the KNN imputation approach to handle the missing values in the dataset related to the independent variables. KNN imputation was chosen over parametric methods as the dataset had a small percentage of missing data (11%), Little’s MCAR test revealed that the missing data mechanism was Missing Completely at Random (MCAR) (p > 0.05), and the relationships between the variables were not strictly linear. ^33, 40^

The raw data were transformed into features that more accurately depicted the underlying issue for the predictive models, improving the accuracy of the unobserved data. Hence, among other feature engineering techniques, label encoding for coding each category of variables as a number and encoding categorical variables into numeric values for nominal variables were carried out. The process of dimension reduction was used to decrease the number of input variables for the predictive model. Having fewer input variables might lead to a simpler predictive model, which can perform better when generating predictions on new information. To ensure that only the most essential dummy variables were included, we reduced the amount of features using RFECV. Since Principal Component Analysis (PCA) is not ideal for one-hot-encoded categorical features. Using those techniques to assess the link between the independent input components and the output variable and selecting the most important independent variables, feature selection and feature extraction was utilized to forecast the target variable. This technique has frequently been used in earlier public health research to identify the factors and/or predictors of different health outcomes. ^30, 41^

This study utilized a standard 80/20 split approach, where 80% of the data were used for training and the remaining 20% were used for model testing. The model was trained using the tenfold cross-validation method, which does not waste much data; this approach is highly beneficial when the number of samples is limited. ^30^ To train the prediction function, K-fold separates all the observations into equal-sized groups of samples termed folds and k-1 folds. The fold that is left out is then used for testing k times repeatedly. ^33^ The average of the results calculated in the loop serves as the k-fold cross-validation performance measure.

**Model selection:** Appropriate models were selected for training after the data were prepared and split into training and testing sets. The task was a binary classifier because the outcome variable was categorical, and suitable classifiers needed to be chosen to make the prediction. Since modern contraceptive use was divided into two mutually exclusive categories (use or not use), the dataset employed in the analysis falls under the category of binary categorization. Hence, in this study, we used six widely used machine learning (ML) algorithms, ^33^ logistic regression (LR), random forest (RF), K-nearest neighbors (KNN), extreme gradient boosting (XGBoost), naive Bayes (NB), and support vector machines (SVMs), to predict determinants of modern contraceptive use among reproductive-age women in Ethiopia and compared the results of the traditional logistic regression model to the results of the best algorithm for identifying the new features influencing the outcome of interest.

**Model training:** The selected classifiers were trained using prepared data after model selection, and their performances were compared via tenfold cross-validation. Following this comparison, the top predictive model was chosen, and it was trained with balanced training data to make the final prediction on hypothetical test data. ^33^

**Model evaluation:** Testing the model’s performance on never-before-seen data that were set aside for this purpose during data splitting can help determine how well the model works after it has been trained. One popular technique for evaluating the effectiveness of a classification model is the confusion matrix, which is a straightforward cross-tabulation of the actual and predicted categories for the outcome variable. The performance criteria, which include the overall accuracy, precision, recall, and F1 score and were employed in this study to evaluate the effectiveness of the selected classifiers, can be calculated using confusion metrics. Additionally, the performance of the ML models was assessed using receiver operating characteristic (ROC) curves, and the value 0.5 = no discrimination. 0.5-0.7 = Poor discrimination. 0.7-0.8 = Acceptable discrimination. 0.8-0.9 = Excellent discrimination. ^41^

**Hyperparameter tuning:** To better understand the possibility of obtaining the optimal values and avoid unnecessary computations for combinations of nonperforming parameters when searching for the optimal parameter settings. Grid search, random search, and Bayesian optimization were used to formulate hyperparameter optimization to improve the speed and quality of the learning process, and we attempted to incrementally adjust the parameters of our model to improve its performance. ^42^

**Figure 1.**
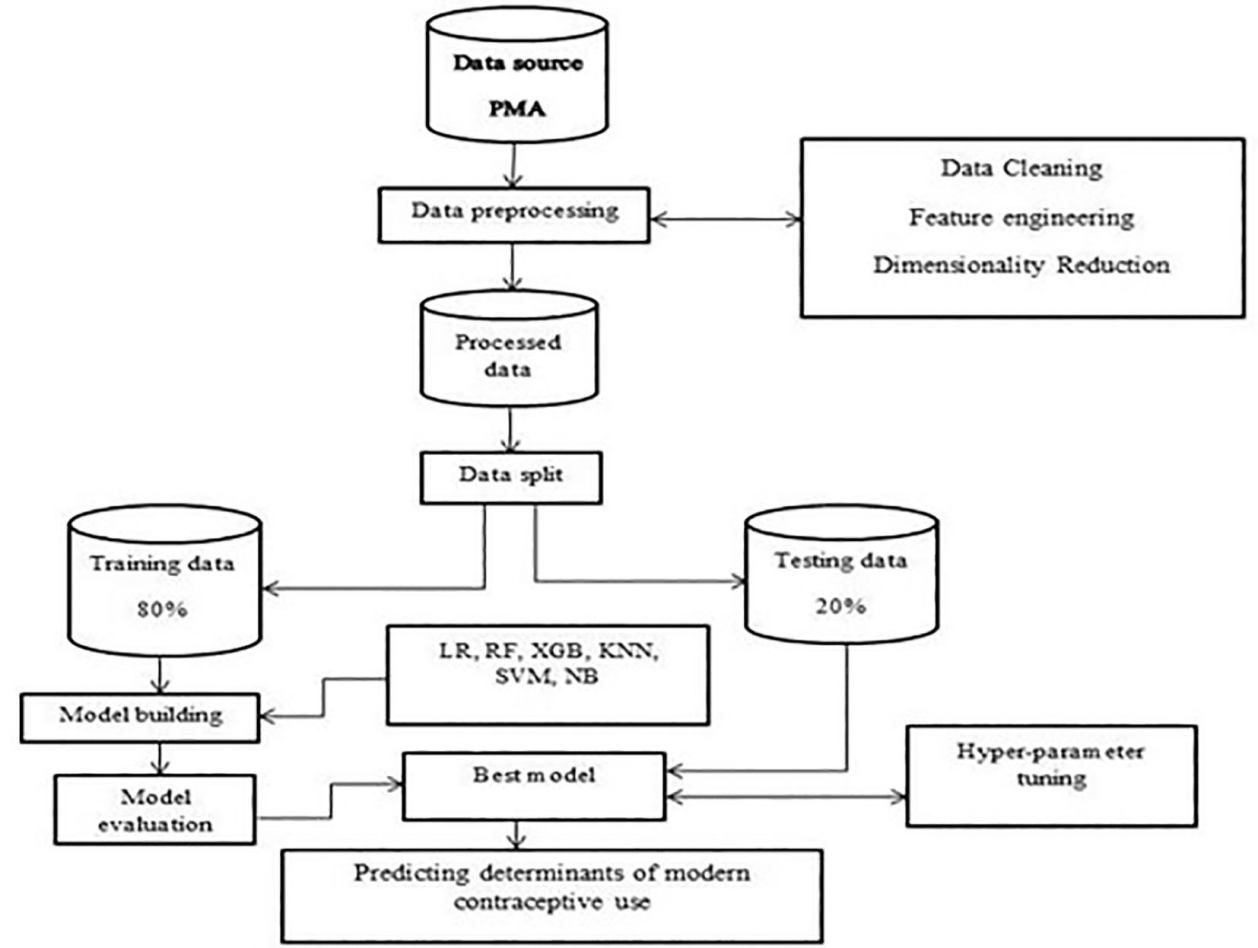
Overview of methodologies.

**Making prediction:** All the aforementioned activities occur in this stage, which is the last stage in machine learning methodology. By using independent variables as a framework, prediction involves estimating the outcome variable. In this instance, modern contraceptive use was established using crucial factors that were discovered along the route. The best-performing classifier with a specified accuracy was used to predict whether a woman would use modern contraceptive services given various factors. The overall workflow of the methodology is shown below ( Figure 1).

## Results

### Socio-demographic and economic characteristics

Most of the women (5177, 56.85%) were rural residents, and 5,326 (58.48%) were aged between 26 and 35 years. The majority of participants was poor (32.48%), had a primary education (54.34%) and had no media access (92.1%). Regarding the regional distribution of respondents, the majority of the women were from Oromia (19.44%), followed by the SNNPR (18.15%), and approximately 17.66% were from Amhara. The remaining regions accounted for 44.75% of the total study population ( Table 2).

**Table 2.** Socio-demographic and economic characteristics of the respondents.

Variable	Categories	Weighted Freq.	Percent (%)
Residence	Urban	1,620	18.33
	Rural	7,217	81.67
Marital status	Married	5,624	63.64
	single	3,213	36.36
Women’s age	15 to 25	2,409	27.54
	26 to 35	5,326	58.48
	36 to 49	1,273	13.98
Region	Tigray	1,196	13.13
	Afar	424	4.66
	Amhara	1,608	17.66
	Oromia	1,770	19.44
	Somali	194	2.13
	Benishangul-Gumuz	289	3.17
	SNNPR	1,653	18.15
	Gambela	350	3.84
	Harari	342	3.76
	Addis	884	9.71
	Dire Dawa	397	4.36
Education level	No education	3,072	34.42
	Primary	4,808	54.34
	secondary and above	1,003	11.24
Wealth index	Poor	2,907	32.48
	Middle	1,263	16.07
	Rich	4,667	51.46
Media access	No	7,916	89.5
	Yes	921	10.5

### Reproductive health and family planning use characteristics of the study participants

Among the total respondents, 6,029 (67.55%) had a history of pregnancy, and approximately 4,769 (70.29%) had started sexual intercourse before the age of 18 years. Approximately 2,544 (43.64%) of the women had never delivered at health facilities. Regarding their partner/husband feelings toward FP use, the majority of the women (3,298; 58.41%) had approved the use of FPs by their husband/partner ( Table 3).

**Table 3.** Reproductive health and family planning service characteristics of the respondents.

Variable	Categories	Weighted Freq.	Percent
Ever been pregnant	No	2,836	32.45
	Yes	6,021	67.55
Age at first sex	Before age 18	4,723	70.29
	After age 18	4,114	29.71
Ever used FP methods	No	4,306	49.19
	Yes	4,531	50.81
Ever delivered in HF	No	2,524	43.64
	Yes	3,313	56.36
Partner/husband feeling about FP	Disapproval	2,626	41.59
	Approved	6,211	58.41
Partner told not to use FP	No	6,505	73.61
	Yes	2,332	26.39

### Modern contraceptive use

**Figure 2.**
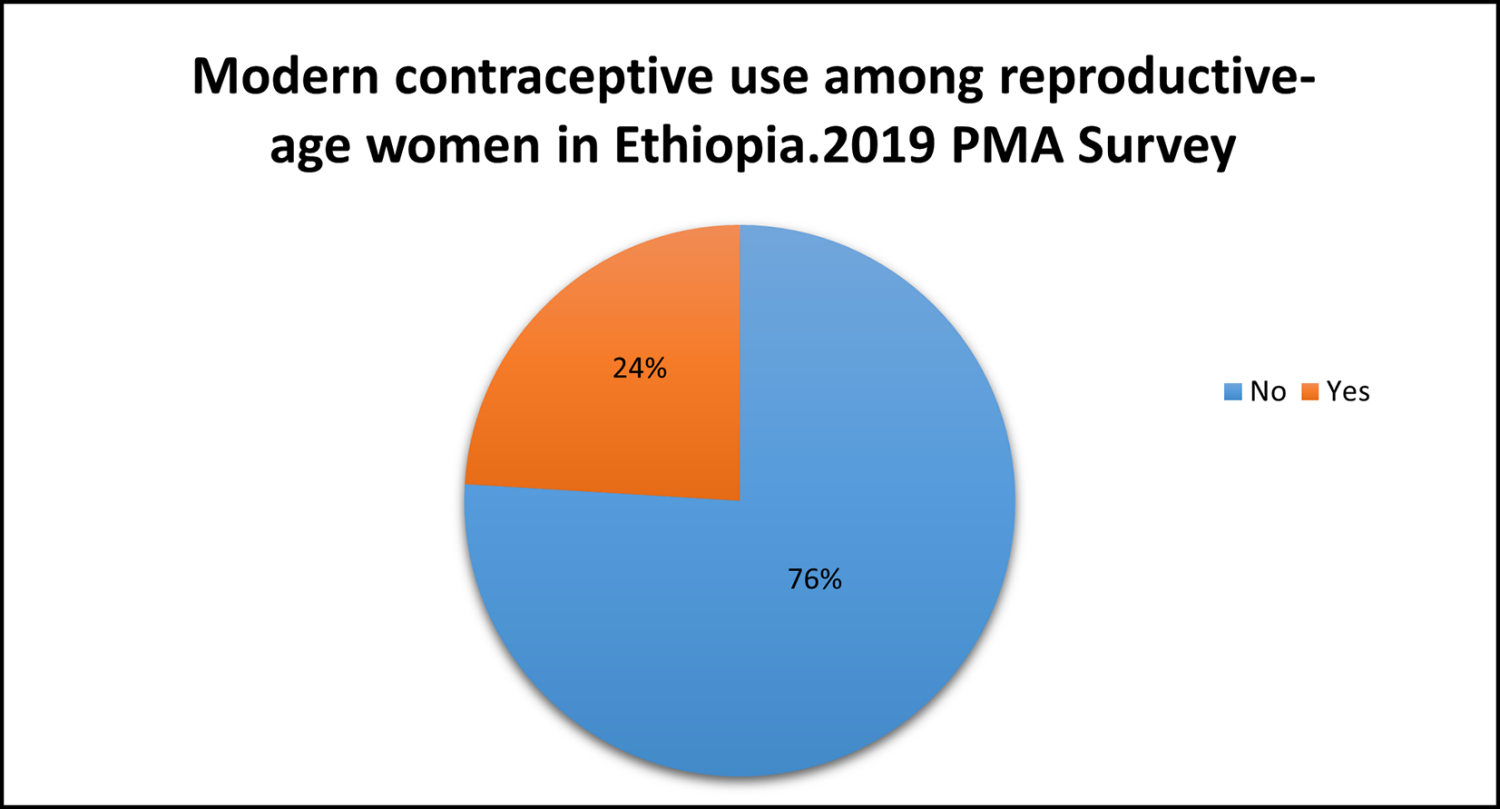
Modern contraceptive use among reproductive-age women in Ethiopia: Evidence from the Performance Monitoring and Accountability (PMA) Survey 2019 dataset.

Among the study participants, only 24% {95% CI (23.1%, 24.9%)} used modern contraceptive methods. Most (1,204, 13.6%) of the modern contraceptive users had a secondary education ( Figure 2).

### Machine learning analysis of modern contraceptive use among reproductive-age individuals in Ethiopia

**Feature selection**

Feature selection is crucial for determining the most important predictors of an outcome variable, similar to how p values and t statistics are used in most traditional statistical methods, to determine which variables are significant. Accordingly, when using an ensemble model to predict an outcome, feature importance measures how significant a feature is on average concerning other features.

**Figure 3.**
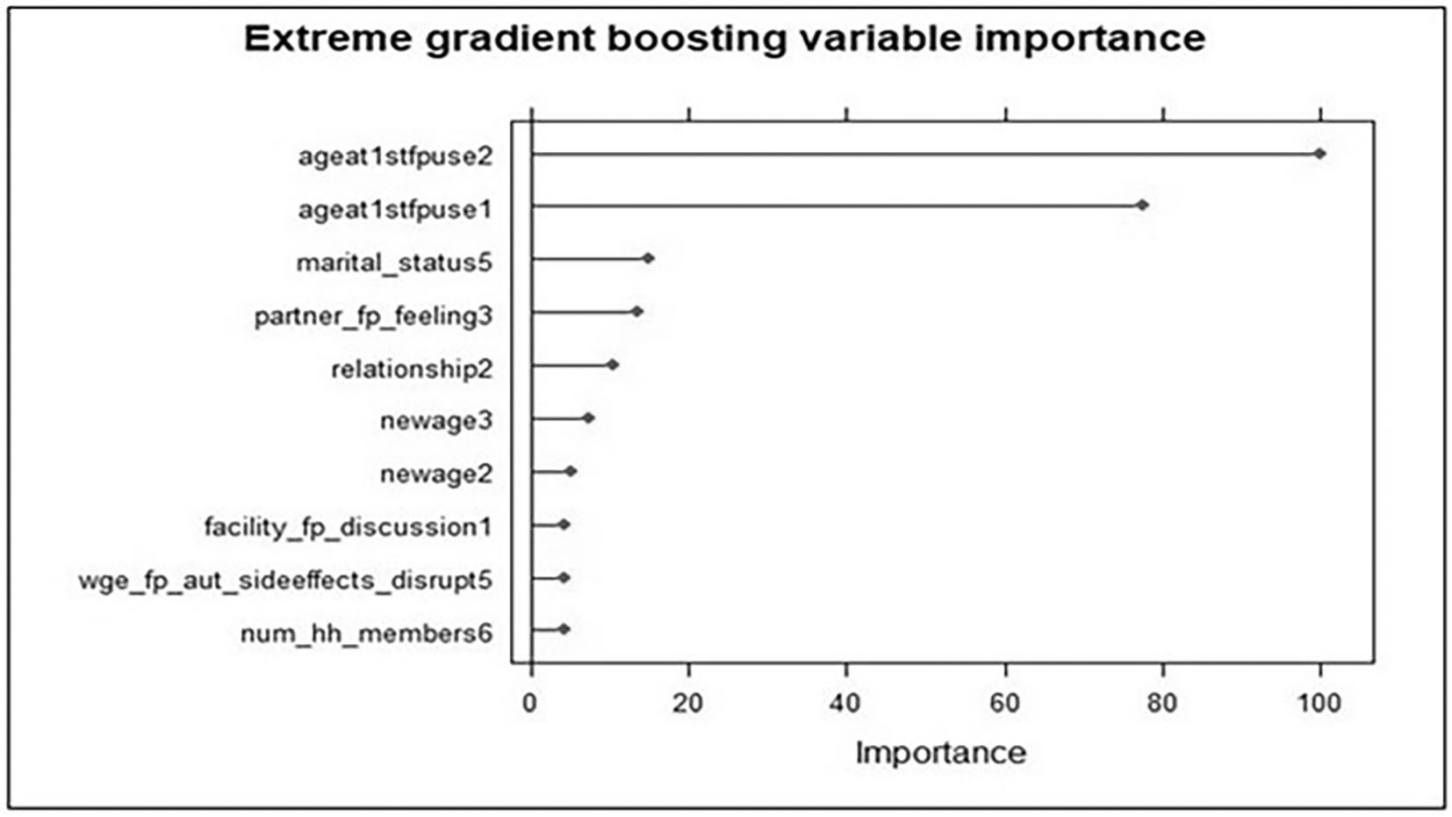
Feature importance plot using the XGBoost model for predictors of modern contraceptive use in Ethiopia.

The most significant predictors of modern contraceptive use according to the extreme gradient boost feature importance results were age at first family planning use (20 years and above), marital status (single), partner/husband feelings about family planning (Approval from partner) and relationship with the head of household (having a wife relationship to the head). In addition, age (36 to 49 years), health care providers advise about the use of FP methods during health facility visit, perception about the FP side effects might disrupt their relationship and household size (5 and above) were other key predictors. The length of the bars on the x-axis, which represents the relative significance of the independent variables in predicting the use of modern contraceptives, shows this. The longer the bar is, the more significant the trait is in determining whether a woman utilizes modern contraception ( Figure 3).

**Model selection**

After the data were prepared and divided into training and testing datasets, as indicated in Table 4, the appropriate models were chosen for the training dataset. To predict the factors that influence the use of modern contraceptives among reproductive-age women in Ethiopia, we used six commonly used machine learning (ML) algorithms, namely, logistic regression (LR), random forest (RF), K-nearest neighbors (KNN), extreme gradient boosting (XGBoost), naive Bayes (NB), and support vector machines (SVMs). We chose the best model from among these machine learning approaches based on its higher level of accuracy ( Table 4).

**Table 4.** Model accuracy metrics for all the models evaluated on the training data.

Algorithms	Minimum	1st Quartile	Median	Mean	3rd Quartile	Maximum
**Logistic Regression**	0.788	0.798	0.802	0.801	0.804	0.809
**Random Forest**	0.795	0.796	0.801	0.800	0.805	0.807
**XGBoost**	0.790	0.803	0.809	0.806	0.812	0.814
**KNN**	0.769	0.778	0.782	0.785	0.790	0.806
**Naive Bayes**	0.758	0.793	0.799	0.797	0.807	0.814
**Linear SVM**	0.792	0.796	0.797	0.800	0.806	0.811

**Figure 4.**
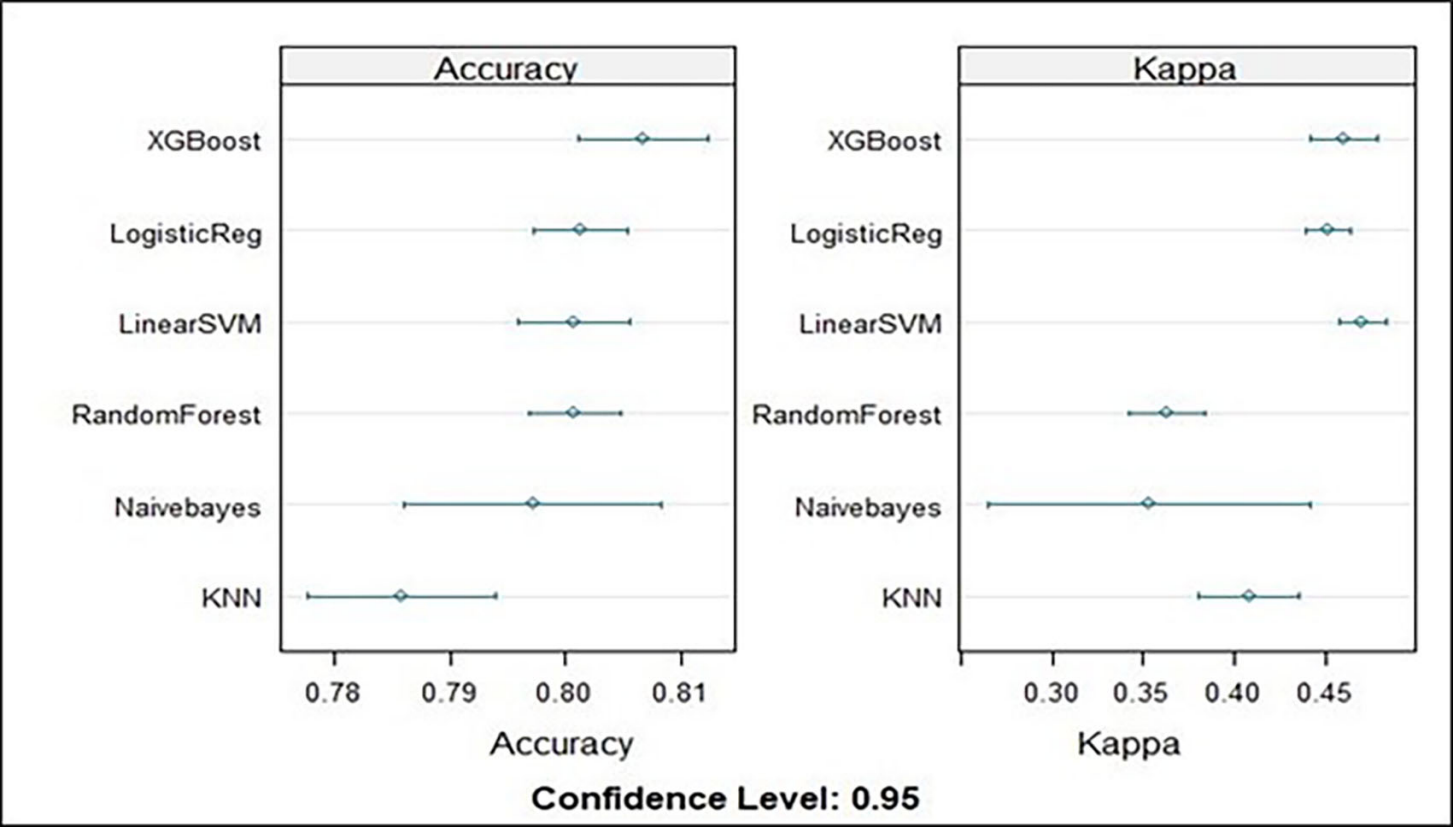
Model accuracy metrics for all the models evaluated on the training data.

A figure based on the mean level of accuracy serves as the representation for the summary models. The extreme gradient boost model therefore had the best model for training the dataset, with a mean accuracy of 80.6% ( Figure 4).

**Prediction of modern contraceptive use**

Using the remaining test data (predictions from unseen test data), the performance of the predictive models for predicting modern contraceptive use was compared using the mean accuracy and mean area under the curve (AUC) of the ML models in stratified tenfold cross-validation.

After all the models were investigated in this study, extreme gradient boosting was found to have the greatest accuracy (81.97%, 95% CI{(79.06%, 82.7%)} as was the ROC area (76.63%), followed by logistic regression with minor difference (80.52%, 95% CI {(78.6%, 82.3%)} and support vector machines (SVMs) (80.41%), 95% CI{(78.48%, 82.2%)}.

The XGBoost model had relatively low specificity (88.67%), which meant that it performed poorly in identifying predictors of modern contraceptive use in Ethiopia but had high sensitivity (66.6%), which meant that it was more accurate in identifying predictors of modern contraceptive use ( Table 5).

**Table 5.** Model accuracy metrics for all the models evaluated on the test data.

Confusion matrix	Logistic Regression		Random Forest		XG Boost		KNN		Naive Bayes		SVM	
	Observed		Observed		Observed		Observed		Observed		Observed	
Predicted	No MCP use	MCP use	No MCP use	MCP use	No MCP use	MCP use	No MCP use	MCP use	No MCP use	MCP use	No MCP use	MCP use
No MCP use	1179	181	1258	249	129	80	175	143	175	143	162	122
MCP use	163	243	84	175	233	494	187	431	187	431	200	452
Metrics	%		%		%		%		%		%	
Accuracy	80.52		81.2		81.97		79.95		79.84		80.41	
95% CI	(78.6, 82.3)		79.3, 83.0)		(79.06, 82.7)		(78.01, 81.8)		(77.89, 81.6)		(78.48, 82.2)	
Sensitivity	57.31		41.27		66.60		56.84		44.34		64.86	
Specificity	87.85		93.81		88.67		87.26		91.06		85.32	
Positive predictive value	59.85		67.83		61.22		58.50		61.04		58.26	
Negative predictive value	86.69		83.48		86.61		86.48		83.81		88.49	
AUC	72.58		70.54		76.63		72.04		69.99		75.08	

**Figure 5.**
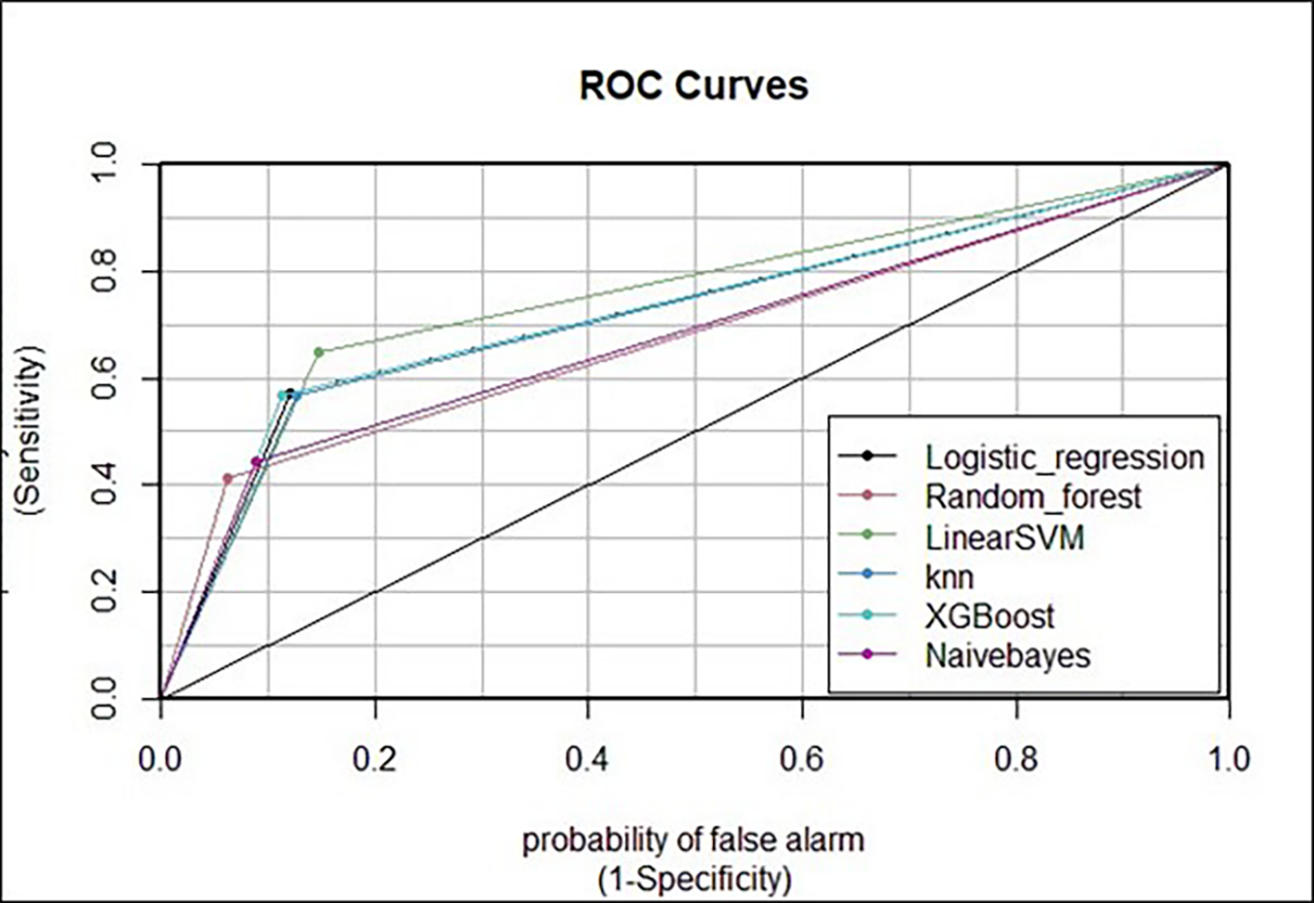
Receiver operating characteristic (ROC) curve of the six models’ AUC percentages for comparison of model predictions on test data.

Visualization of the receiver operating characteristic (ROC) curve was performed. Among the six machine learning models employed in this study, the curve of the extreme gradient boost model had the highest percentage of AUC values, indicating that it is the best at classifying the use or nonuse of modern contraceptive methods among reproductive-age women in Ethiopia. Moreover, this best model represented an acceptable range of AUC values (76.63%) ( Figure 5).

## Discussion

In this study, a weighted sample of 8837 women of reproductive age was employed for the final analysis, which was limited to secondary data from the PMA Ethiopia 2019 Cross-sectional Household and Female Survey. The use of modern contraceptive methods was found to be extremely low (24%), ^43^ which is comparable to the findings of earlier studies carried out in Ethiopia. These earlier studies revealed that the use of modern contraceptive methods was 31.7% in rural Dembia District, northwestern Ethiopia ^7^; 11.0% in the surrounding Peasant Association of Gondar Town ^7^; 38.3% in Mojo Town, southern Ethiopia ^44^; and 67.4% in Hosanna. ^45^ The possible reason could be an increased expansion of government and private health institutions, including health posts, as well as the communication of information by health extension workers and various nongovernmental organizations (NGOs). The difference might also be due to differences in awareness of modern contraceptive methods.

To identify determinants of modern contraceptive use, each algorithm was trained on 80% of the total instances through random sampling, and its effectiveness was tested on 20% of the total instances through random sampling. Six widely used machine learning (ML) algorithms, logistic regression (LR), random forest (RF), K-nearest neighbors (KNN), extreme gradient boosting (XGBoost), naive Bayes (NB), and support vector machines (SVMs), were included in the study to predict determinants of modern contraceptive use among reproductive-age women in Ethiopia.

The performance of the predictive models in predicting determinants of modern contraceptive use was assessed using the remaining tested data (predictions from unseen test data) and compared against the mean accuracy and mean area under the curve (AUC) of the ML models in stratified tenfold cross-validation. Accordingly, extreme gradient boosting had the highest accuracy (81.97%), 95% CI (79.06%, 82.7%) and area under the ROC curve (76.63%). The performance of this model is much better than that of studies conducted on the prediction of contraceptive discontinuation among reproductive-age women in Ethiopia using the Ethiopian Demographic and Health Survey 2016 dataset. These studies used the random forest model as the best predictive model, with an accuracy of 68% and an ROC of 74% based on a tenfold cross-validation score on balanced training data ^46^; additionally, another study conducted in Ethiopia also found the random forest model to be the best machine learning model for predicting nutritional status for children under five years of age using EDHS data, with an accuracy and AUC of 68.2% and 0.76, respectively. ^47^ These results, however, are lower than those of an Indonesian study in which AdaBoost was identified as the most accurate model for predicting the duration of contraceptive use, for which the accuracy was 85.1%. The size of the dataset used to develop the model may be the cause of this mismatch. The Indonesian study employed 39,594 records, whereas this study used only 8837 records, allowing the model to train more effectively and make predictions with greater accuracy.

Specific characteristics related to the use of modern contraceptives in Ethiopia that can be used as intervention targets were compared, identified, and recognized with the aid of machine learning methods. The extreme gradient boosting (XGB) and support vector machine models have the highest prediction power among the constructed predictive models compared to other machine learning classifier models, such as the RF and KNN models. According to the extreme gradient boost feature importance results, the variables were age at first family planning use, marital status, partner/husband feelings about family planning and relationship with the head of household. In addition, age and health care providers spoke about FP methods at health facility visits; if I use FPs, side effects might disrupt my relationship, and household size is also an important predictor of modern contraceptive use. This study is roughly in line with previous findings.

Age at first family planning use (20-30 years and above) was the first significant characteristic of modern contraceptive use among reproductive-age women in Ethiopia. This finding was consistent with that of a study performed in Bangladesh, southern Ethiopia, Nigeria, and the Democratic Republic of the Congo, which revealed that as women’s age increased from 15 to 34, the likelihood that they would use contraceptives increased. ^48– 51^ The most likely explanation is that in rural settings, this age range is when most women are involved in various activities to take care of their household’s requirements, leading them to wish to space their pregnancies. Therefore, they favor the use of contraceptive techniques. The other factor might be that people in this age group now have women’s forum associations to debate the topic, as well as greater experience sharing from colleagues and neighbors. As a result, their utilization rate could increase. These results, however, did not align with research carried out in Mojo town, ^44^ and a study performed in Kerman, ^52^ Iran, revealed that people who used modern contraceptives had a younger mean age than people who did not. Differences in socio-demographic characteristics and durations may also explain the differences.

The marital status of reproductive-age women in Ethiopia was one of the other most significant factors for predicting modern contraception use. This conclusion was consistent with the findings of studies conducted in Tanzania and Gondar, Ethiopia, which showed that married women were more likely to use modern contraceptives than unmarried (single, widowed, or divorced) women were. ^6, 7^ The outcome highlights the significance of male involvement in reproductive health issues, such as fertility and contraception, as well as couples’ motivation through education. Counseling and FP education should encourage couples to share their fertility concerns with one another.

Couples’ desire to have a/another child was also the most important feature of modern contraceptive use in predicting reproductive-age women in Ethiopia. This finding is consistent with the findings of studies conducted on predictors of modern contraceptive method use among married women of reproductive age in Western Ethiopia and elsewhere, which showed that those respondents who did not express future desire for children were 2.6 times more likely to utilize modern contraceptives during the study period. ^20, 21^ It was obvious that women who desired children were not ready to use contraceptives.

The most significant aspect of modern contraceptive use predictions among Ethiopian women of reproductive age was the partner/husband’s attitude toward family planning. These findings are consistent with research conducted in Gondar, Ethiopia. ^7^ This may be attributable to the discussion that can lead to an appropriate decision regarding the selection and use of FP methods, and the fact that the discussion was present in rural areas suggested that there may be a high level of knowledge regarding FP methods.

The most significant aspect of modern contraceptive use prediction was the discussion of FP techniques by healthcare personnel during health facility visits. This result is in line with the findings of other studies performed elsewhere that emphasize the need to promote contraceptive use after delivery by utilizing the prenatal period as a window of opportunity. Effective contraception counseling included in comprehensive ANC not only boosts client satisfaction and prenatal care quality but may also lead to an increase in postpartum contraceptive use. ^53, 54^ There is evidence suggesting that health care workers could be trained/retrained to provide more effective FP services through group health education sessions, the distribution of simple educational material to postpartum FPs, individualized counseling and the initiation of chosen contraceptive methods. ^55, 56^

### Limitations and strengths of the study

✓ A key limitation of this study is the regional imbalance of the sample, with majority of participants being from rural areas, which may limit the generalizability of the findings to more urban populations.
✓ Due to their black-box nature, supervised machine learning algorithms do not have coefficients such as odds ratios or incident rate ratios. Therefore, the strength and direction of associations are unknown, additionally absence of external validation and risk of over fitting are also the major limitation of this study.
✓ Moreover, the current study emphasized mothers-related attributes more. Fathers’-related attributes, such as fathers’ education and income level, were missed; hence, future researchers recommend conducting similar studies by addressing the limitations of this study.

## Conclusion and Recommendations

In this study, the utilization of modern contraceptive methods was found to be extremely low. Six widely accepted machine learning algorithms have been used to predict determinants of modern contraceptive use in Ethiopia. Different confusion matrices were used to compare the candidate supervised machine learning algorithms. Based on the predictive model result results, extreme gradient boosting (XGB) was the best performing model and age at first family planning use, marital status, partner/husband feelings about family planning and relationships with the head of household, women’s age, having discussions with healthcare providers about FP methods at health facility visits, and household size were important predictors of modern contraceptive use. The use of modern contraceptives is therefore expected to increase with effective contraceptive counseling during ANC/PNC follow-up on family planning use and increasing partner discussions on FP. Enhancing contraceptive counseling techniques concerning the age at which family planning is used for the first time and the engagement of men in FP should also be investigated. It was also necessary to consider enabling women to choose their methods through spousal discussion and providing health information to modify traditional attitudes around the number of children, which was seen as beneficial for the family.

Furthermore, Future study should employ causal inference designs (randomized controlled trials, longitudinal analyses, or quasi-experimental) to validate and clarify the predictive associations identified in this analysis and to check whether variable identified in this predictive result can causally influence contraceptive use.

### Ethical approval and consent to participate

Ethical clearance and consent was not necessary for this study since it was based on publicly available data sources. Permission to use the data was granted by the PMA Ethiopia’s survey project through legal registration.

## Consent for publication

Not applicable.

## Patient and public participation

Not applicable.

## Author’s contributions

JBA, Conceived and designed the study; analysis, interpreted the result and wrote the paper. AAA, SDK, ADW and DNM made significant contributions to the work reported; contributed to the acquisition of data, contributed to all these areas; participated in drafting, revising or critically reviewing the article; and agreed to be accountable for all aspects of the work. All the authors read and approved the final manuscript.

## Data Availability

The datasets analyzed in the current study are available in the Performance Monitoring for Action repository, https://www.pmadata.org/data/request-access-datasets. The full datasets analysed in the current study are available in the Performance Monitoring for Action (PMA) Ethiopia cross-sectional household and women’s survey. (DOI: https://www.pmadata.org/data/request-access-datasets). The project comprises three key components: annual cross-sectional surveys of women aged 15–49, longitudinal studies tracking pregnant women and new mothers, and yearly service delivery point surveys assessing health facilities. Data are available under the terms of the Creative Commons Attribution 4.0 International license (CC-BY 4.0).
