# Supplementary material for: Predicting determinants of modern contraceptive use among reproductive-age women in Ethiopia using machine learning algorithms: Evidence from the Performance Monitoring and Accountability (PMA) Survey 2019 dataset
Source: F1000Res. 2025 Nov 28;14:99. Originally published 2025 Jan 17. [Version 2] doi: 10.12688/f1000research.156316.2 (PMC12686783; doi:10.12688/f1000research.156316.2)
Supplement: Peer review history

# Reviewer report: Reviewer response for version 2

**DOI:** 10.5256/f1000research.191315.r438239

**Published:** 2026-01-16

**Authors:** Nora Müller, Hao-Ting Chan

**Affiliations:** 1 DRS, GESIS –Leibniz-Institute for the Social Sciences, Mannheim, Mannheim, Germany; 2 CSS, GESIS - Leibniz Institute for the Social Sciences Cologne (Ringgold ID: 28363), Cologne, North Rhine-Westphalia, Germany

**Review**

**F1000 Research**

Title of reviewed paper (R1):

*Predicting determinants of modern contraceptive use among reproductive-age women in Ethiopia using machine learning algorithms: Evidence from the Performance Monitoring and Accountability (PMA) Survey 2019 dataset*

**Overall evaluation**

 The manuscript addresses an important topic — modern contraceptive use among reproductive-age women in Ethiopia — using data from the 2019 PMA survey and a set of supervised machine learning (ML) algorithms. While the revised version improves clarity in some methodological aspects compared to version 1, substantial conceptual and analytical weaknesses remain. Most importantly, the manuscript does not clearly articulate a research gap or a concrete scientific contribution, and it overstates the advantages of machine learning without sufficiently demonstrating them in the empirical analysis.

 At present, the paper reads more as an application of standard ML techniques to a well-studied outcome rather than as a contribution that advances theory, methodology, or substantive knowledge in a clearly defined way.

**Major comments**

1. *No Clearly Identified Research Gap*

The manuscript does not clearly identify or articulate a specific research gap. While the introduction repeatedly claims limitations of “traditional regression models” and motivates the use of machine learning, this motivation remains generic and underdeveloped. It is not made explicit:

- what is missing in the existing literature on modern contraceptive use,
- why existing regression-based studies are insufficient for the research question at hand,
- or what new knowledge the present study aims to generate beyond prior findings.

*Suggestions:*

 The authors should explicitly state a research gap (or gaps) early in the introduction and explain how the chosen design and methods are intended to address it.

1. *No Clearly Stated Contribution*

Relatedly, the manuscript does not clearly state its scientific contribution. The identified predictors (e.g., partner approval, age, counseling by health workers, household size) are well-established in the existing literature on contraceptive use in Ethiopia and other Sub-Saharan African contexts. Thus, the contribution cannot lie in the discovery of new determinants.

 At present, it remains unclear whether the intended contribution is:

- methodological (demonstrating the usefulness of ML),
- applied (supporting program targeting or risk profiling),
- or purely comparative (benchmarking different algorithms).

Without explicitly stating this, the manuscript risks overstating its novelty.

*Suggestions:*

 The authors should clearly articulate what the paper contributes beyond prior studies and explicitly acknowledge that the contribution is predictive and applied, rather than causal or theoretical.

1. *Weak Justification for the Use of Machine Learning*

The arguments provided for the advantages of machine learning over traditional regression models remain weak and largely rhetorical:

- Partially False: “…Logistic regressions, assume linear relationships...”. LR does not assume a linear relationship between the outcome and predictors. Rather, it assumes a linear relationship on the logit scale (the log-odds scale). (Hosmer et al. 1989; Yay 2023)
- Misleading: “…ML algorithms can efficiently handle nonlinear associations, multicollinearity…”. ML face the same challenge, they struggle with multicollinearity, though some handle it differently through regularization or feature selection methods. Both LR and ML learning approaches implement techniques to address correlated predictors. (Levy and O’Malley 2020)
- Empirical inconsistency: As shown in Figure 4, logistic regression is the second best performing model, with an accuracy difference of only 0.5 compared with the top performing model. This marginal improvement does not provide strong empirical support for favoring machine learning approaches, particularly given their increased complexity and reduced interpretability.

Claims regarding nonlinear relationships, multicollinearity, and higher-order interactions are stated, but not convincingly demonstrated or exploited in the analysis.

 In contrast to stronger methodological discussions in recent literature, the manuscript does not explain:

- which specific limitations of logistic regression are most relevant in this context,
- why these limitations are substantively important for understanding contraceptive use,
- or how ML meaningfully overcomes them in practice.

*Suggestions:*

 The justification for ML should be strengthened and made more concrete, or alternatively, the claims regarding its superiority should be substantially toned down. See Sun (2024) for valuable arguments for the advantages of ML models in explorative studies.

1. *Claimed Ability to Capture Interactions Is Not Empirically Used*

One of the main stated advantages of machine learning models is their ability to automatically capture interaction effects. However, the manuscript does not actually examine or interpret any interactions, nor does it attempt to show that interactions play a meaningful role in prediction.

 This raises a critical question: If interactions are a key justification for ML, why are they neither explored nor discussed?

*Suggestion:*

 The authors should either:

- explicitly analyze and interpret interaction structures (e.g., via SHAP interaction values or partial dependence plots), or
- refrain from presenting interaction modeling as a key advantage of their approach.

 

1. *Outcome Prevalence and Model Evaluation*

Modern contraceptive use has a relatively low prevalence (24%), resulting in a pronounced class imbalance. In such a setting, overall accuracy is a limited and potentially misleading performance metric.

 Although sensitivity and specificity are reported, the evaluation would benefit from:

- balanced accuracy,
- precision – recall curves or PR-AUC,
- F1 score, a harmonic mean of precision and recall
- and calibration measures.

This is particularly important given the policy-oriented interpretation of the findings.

1. *Inconsistent Interpretation of “Most Important” Predictors*

The discussion contains internally inconsistent statements about which predictors are most important: On page 15, the manuscript states that “the most significant aspect of modern contraceptive use predictions … was the partner/husband’s attitude toward family planning.” On page 16, it states that “the most significant aspect … was the discussion of FP techniques by healthcare personnel during health facility visits.”

 It is unclear how “most significant” is defined here and on what basis these claims are made.

*Suggestions:*

 If relative importance is central to the contribution, it should be quantified and presented systematically. In this regard, SHAP values or permutation-based importance measures would be highly appropriate, as they allow for transparent comparison of predictor contributions and improve interpretability.

  

1. *Feature Importance Visualization and Labeling (Figure 3)*

Figure 3 suffers from several issues:

- The labeling is difficult to read and not self-explanatory.
- Two age-related variables appear at the top of the importance ranking, but their conceptual distinction is not discussed.
- The figure does not allow the reader to clearly assess relative importance across predictors.

*Suggestions:*

 Improve the readability and interpretability of Figure 3 (clear labels, consistent naming, possibly grouping related variables). A SHAP summary plot would substantially improve clarity.

**Minor comments**

- Statements referring to model “specificity” as being “low” despite values around 88% are confusing and conceptually inaccurate.
- The language occasionally reverts to causal phrasing (“expected to increase”), which should be avoided in a predictive study.
- Software environments and package versions should be reported consistently.

**References**

 refer reference no. 1, 2, & 3

Is the work clearly and accurately presented and does it cite the current literature?

Partly

If applicable, is the statistical analysis and its interpretation appropriate?

Partly

Are all the source data underlying the results available to ensure full reproducibility?

Partly

Is the study design appropriate and is the work technically sound?

Yes

Are the conclusions drawn adequately supported by the results?

Partly

Are sufficient details of methods and analysis provided to allow replication by others?

Partly

Reviewer Expertise:

family sociology, machine-learning, contraceptive behavior

We confirm that we have read this submission and believe that we have an appropriate level of expertise to confirm that it is of an acceptable scientific standard, however we have significant reservations, as outlined above.

## References

[1] : Don’t dismiss logistic regression: the case for sensible extraction of interactions in the era of machine learning. *BMC Medical Research Methodology* .2020;20(1) : 10.1186/s12874-020-01046-3 10.1186/s12874-020-01046-3 32600277 PMC7325087

[2] : Assessment of the factors that affect fast-track or early extubation following pediatric cardiac surgery: Logistic regression in clinical studies. *Turkish Journal of Thoracic and Cardiovascular Surgery* .2023;31(1) : 10.5606/tgkdc.dergisi.2023.98550 8-10 10.5606/tgkdc.dergisi.2023.98550 PMC1001297736926160

[3] : Supervised machine learning for exploratory analysis in family research. *Journal of Marriage and Family* .2024;86(5) : 10.1111/jomf.12973 1468-1494 10.1111/jomf.12973

# Reviewer report: Reviewer response for version 2

**DOI:** 10.5256/f1000research.191315.r436929

**Published:** 2025-12-08

**Authors:** Lotenna Olisaeloka

**Affiliations:** 1 The University of British Columbia, Vancouver, British Columbia, Canada

I now approve the revised version.

Is the work clearly and accurately presented and does it cite the current literature?

Yes

If applicable, is the statistical analysis and its interpretation appropriate?

Partly

Are all the source data underlying the results available to ensure full reproducibility?

Yes

Is the study design appropriate and is the work technically sound?

Yes

Are the conclusions drawn adequately supported by the results?

Partly

Are sufficient details of methods and analysis provided to allow replication by others?

Partly

Reviewer Expertise:

NA

I confirm that I have read this submission and believe that I have an appropriate level of expertise to confirm that it is of an acceptable scientific standard.

# Reviewer report: Reviewer response for version 1

**DOI:** 10.5256/f1000research.171606.r415900

**Published:** 2025-11-05

**Authors:** Lotenna Olisaeloka

**Affiliations:** 1 The University of British Columbia, Vancouver, British Columbia, Canada

Summary of the Article

 The manuscript analyzes data from the 2019 Performance Monitoring and Accountability (PMA) survey to identify predictors of modern contraceptive use among Ethiopian women of reproductive age. Using a weighted sample of 8,837 participants, the authors applied six supervised machine-learning algorithms and compared their predictive accuracy.

 XGBoost reportedly achieved the highest performance (accuracy of approx. 82% and AUC of 0.77). The authors conclude that age at first family-planning use, marital status, partner approval, women’s age, household size, and health-provider counseling were the strongest predictors of contraceptive use. The authors recommend improving contraceptive counseling and fostering partner communication to increase modern contraceptive uptake. 

 The topic is very relevant to reproductive health in low-resource settings, especially Sub-Saharan Africa, and applying ML methods represents an advanced analytical approach. However, several issues concerning conceptual framing/scope, methodology, and interpretation must be addressed to improve the rigor and presentation of this paper. 

 1. Clarity and Literature Grounding

 The article is generally readable and references pertinent literature, but several statements need clarification or correction for accuracy: 

1. Quantitative claims such as “308 million unwanted pregnancies saved” are ambiguous and lack a clearly defined time frame and source context. Consider rephrasing and using well-supported, up-to-date references to enhance the credibility of your argument. 
2. The paper would benefit from the inclusion of a definition for “modern contraceptives” (with examples) to help delineate the study scope. 
3.  Some background paragraphs mix causes of low contraceptive use with consequences (e.g., high fertility, maternal mortality). Reordering and clearer transitions would improve flow and logic. 
4. The rationale for adopting ML instead of traditional regression models is underdeveloped. Furthermore, the phrase “problems involving multidisciplinary relationships between variables and many factors” is quite unclear in the sentence context. Consider explaining the analytical challenges (e.g., nonlinearities, multicollinearity, interaction effects) that ML was expected to handle. 
5. Explain all abbreviations (e.g. KNN) on the first use and include them all in the abbreviation list. 

2. Study Design and Data Description

 Using PMA-Ethiopia data is appropriate, but the paper omits crucial methodological details that affect validity and reproducibility:

1. Sampling methodology: While the study mentions weighting, it does not sufficiently explain the PMA survey design and whether stratification or clustering were incorporated and accounted for. Indicating these would help readers better understand the survey’s sampling procedure, how weights were applied, and help confirm national representativeness. 
2. Inclusion/exclusion criteria: The process by which the final analytical sample was derived is unclear. Please specify which respondents were included and which were excluded if any, from the initial sample. A simple participant-flow diagram could help clarify this. 
3. Operationalization of variables: For the predictor variable, please define what constructs (such as wealth index and media access) mean or how they were derived from the survey. For the outcome variable, also clarify whether “modern contraceptive use” refers to current or ever use, and how the dichotomous variable was generated from PMA items. 

3. Data Processing and Analytical Methods

1. The manuscript alternates between using Python (in Google Colab) and R with caret package for data analysis. For transparency and reproducibility purposes, clearly specify which environment and packages were actually used. 
2. The description of feature selection and dimensionality reduction process appear too general. Consider identifying the exact technique (e.g., information gain, recursive feature elimination) and justifying its choice.
3. Handling of missing data: There was no discussion of missing data in the survey and how it was handled.  

4. Results and Statistical Interpretation

1. The descriptive statistics are clear, but the urban–rural and regional imbalance of the sample (82% rural) may reflect issues with the survey/sampling technique and could limit result generalizability. 
2. The feature-importance plot (Figure 3) uses coded variable names that are difficult to interpret. Consider replacing with plain-language labels. 
3. Minor differences in accuracy across algorithms (≈ 80–82%) suggest limited added value of ML over logistic regression, which should be acknowledged. 

5. Discussion and Conclusions

 The discussion appropriately references comparable studies but often blurs the distinction between prediction and causation. Statements implying that identified features determine contraceptive use are not supported by a predictive design. Interpretations such as “use is therefore expected to increase with counseling and partner discussion” require caution as they are policy implications, not model findings. Instead, consider framing results as predictive associations that may inform hypothesis generation for future causal research. 

 Regional and contextual explanations are underdeveloped. For instance, large urban–rural and regional disparities in contraceptive use could have been better discussed in relation to service availability or sociocultural norms. 

 The limitations section acknowledges the “black-box” nature of ML but should also consider the absence of external validation and risk of overfitting.

Is the work clearly and accurately presented and does it cite the current literature?

Yes

If applicable, is the statistical analysis and its interpretation appropriate?

Partly

Are all the source data underlying the results available to ensure full reproducibility?

Yes

Is the study design appropriate and is the work technically sound?

Yes

Are the conclusions drawn adequately supported by the results?

Partly

Are sufficient details of methods and analysis provided to allow replication by others?

Partly

Reviewer Expertise:

Public Health, Global Health, Digital Health, Artificial Intelligence.

I confirm that I have read this submission and believe that I have an appropriate level of expertise to confirm that it is of an acceptable scientific standard, however I have significant reservations, as outlined above.

## References

[1] Adem JB, Kebede SD, Walle AD and Mamo DN. Predicting determinants of modern contraceptive use among reproductive-age women in Ethiopia using machine learning algorithm: Evidence from the Performance Monitoring and Accountability (PMA) Survey 2019 dataset [version 1; peer review: 1 approved]. F1000Research 2025, 14:99 (https://doi.org/10.12688/f1000research.156316.1).10.12688/f1000research.156316.2PMC1268678341376854

# Response

**Published:** 2025-11-12

**Authors:** Jibril Bashir Adem

**Affiliations:** Public Health, Arsi University, Asella, Oromia, Ethiopia

**Summary of the Article**

 This manuscript analyzes data from the 2019 Performance Monitoring and Accountability (PMA) survey to identify predictors of modern contraceptive use among Ethiopian women of reproductive age. Using a weighted sample of 8,837 participants, the authors applied six supervised machine-learning algorithms and compared their predictive accuracy.

 XGBoost reportedly achieved the highest performance (accuracy of approx. 82% and AUC of 0.77). The authors conclude that age at first family-planning use, marital status, partner approval, women’s age, household size, and health-provider counseling were the strongest predictors of contraceptive use. The authors recommend improving contraceptive counseling and fostering partner communication to increase modern contraceptive uptake.

 The topic is very relevant to reproductive health in low-resource settings, especially Sub-Saharan Africa, and applying ML methods represents an advanced analytical approach. However, several issues concerning conceptual framing/scope, methodology, and interpretation must be addressed to improve the rigor and presentation of this paper.

**1. Clarity and Literature Grounding**

 The article is generally readable and references pertinent literature, but several statements need clarification or correction for accuracy:

- Quantitative claims such as “308 million unwanted pregnancies saved” are ambiguous and lack a clearly defined time frame and source context. Consider rephrasing and using well-supported, up-to-date references to enhance the credibility of your argument.

**Author response: **Thank you very much for this comment; we revised the sentence to improve its clarity.

- The paper would benefit from the inclusion of a definition for “modern contraceptives” (with examples) to help delineate the study scope.

**Author response: **We included the definition and overview of modern contraceptives in the manuscript. Thank you

- Some background paragraphs mix causes of low contraceptive use with consequences (e.g., high fertility, maternal mortality). Reordering and clearer transitions would improve flow and logic.

**Author response: **We revised the sentences about the advantages of effective modern contraceptive utilization for improving maternal mortality, focusing on reordering and clearer transitions. Thank you

  

- The rationale for adopting ML instead of traditional regression models is underdeveloped. Furthermore, the phrase “problems involving multidisciplinary relationships between variables and many factors” is quite unclear in the sentence context. Consider explaining the analytical challenges (e.g., nonlinearities, multicollinearity, interaction effects) that ML was expected to handle.

**Author response: **We revised the manuscript by integrating your important suggestions about explaining the analytical challenges (e.g., nonlinearities, multicollinearity, interaction effects) that ML was expected to handle. Thank you

 5. Explain all abbreviations (e.g., KNN) on the first use and include them all in the abbreviation list.

**Author response: **We included an explanation of each word's first abbreviation throughout the text. Thank you

**2. Study Design and Data Description**

 Using PMA-Ethiopia data is appropriate, but the paper omits crucial methodological details that affect validity and reproducibility:

- Sampling methodology: While the study mentions weighting, it does not sufficiently explain the PMA survey design and whether stratification or clustering was incorporated and accounted for. Indicating these would help readers better understand the survey’s sampling procedures and how weights were applied, and help confirm national representativeness.

**Author response: **We included a detailed description of the survey, including design and stratification/clustering-related information, in the manuscript. Thank you

- Inclusion/exclusion criteria: The process by which the final analytical sample was derived is unclear. Please specify which respondents were included and which were excluded, if any, from the initial sample. A simple participant-flow diagram could help clarify this.

**Author response: **We revised the manuscript by including the inclusion and exclusion criteria of the study. Thank you

- Operationalization of variables: For the predictor variable, please define what constructs (such as wealth index and media access) mean or how they were derived from the survey. For the outcome variable, also clarify whether “modern contraceptive use” refers to current or ever use, and how the dichotomous variable was generated from PMA items.

**Author response: **We included a definition and measurements of outcome and predictor variables of the study in the revised manuscript. Thank you

 3. **Data Processing and Analytical Methods**

 1. The manuscript alternates between using Python (in Google Collab) and R with the caret package for data analysis. For transparency and reproducibility purposes, clearly specify which environment and packages were used.

**Author response: **We revised the sentence to indicate the use of R software version 4.4.3 for variable extraction and imputation and Python 3.11 for building machine learning (ML) algorithms. Thank you

 2. The description of the feature selection and dimensionality reduction process appears too general. Consider identifying the exact technique (e.g., information gain, recursive feature elimination) and justifying its choice.

**Author response: **We have included the use of recursive feature elimination and its justification in the revised manuscript. Thank you

1. Handling of missing data: There was no discussion of missing data in the survey and how it was handled. 

**Author response: **We have included detailed explanations of the missing data handling method with its justifications in the revised manuscript. Thank you

**4. Results and Statistical Interpretation**

 1.         The descriptive statistics are clear, but the urban–rural and regional imbalance of the sample (82% rural) may reflect issues with the survey/sampling technique and could limit the results’ generalizability.

**Author response: **We included the regional imbalance of the sample and its impact on the generalizability of the findings as a key limitation of this study in the limitations section. Thank you

 2. The feature-importance plot (Figure 3) uses coded variable names that are difficult to interpret. Consider replacing them with plain-language labels.

**Author response: **We have removed the coded variable, as the variable's description was already indicated in Table 1. Thank you

 3. Minor differences in accuracy across algorithms (≈ 80–82%) suggest limited added value of ML over logistic regression, which should be acknowledged.

**Author response: **We acknowledged the minor difference between the two models in the revised manuscript. Thank you

**5. Discussion and Conclusions**

 1. The discussion appropriately references comparable studies but often blurs the distinction between prediction and causation. Statements implying that identifiable features determine contraceptive use are not supported by a predictive design. Interpretations such as “use is therefore expected to increase with counseling and partner discussion” require caution, as they have policy implications, not model findings. Instead, consider framing results as predictive associations that may inform hypothesis generation for future causal research.

**Author response: **We indicated the point that the results of this study are predictive association, rather than causal association, in the revised manuscript. Thank you

 2. Regional and contextual explanations are underdeveloped. For instance, large urban–rural and regional disparities in contraceptive use could have been better discussed in relation to service availability or sociocultural norms.

**Author response: **Regional disparity of the participants was not found to be among the most important predictors by the model; thus, we are unable to discuss it in relation to service availability or sociocultural norms. However, specific personal characteristics like religion and educational level were found to be the most important, and they were discussed accordingly. Thank you

 3. The limitations section acknowledges the “black-box” nature of ML but should also consider the absence of external validation and risk of overfitting.

**Author response: **We revised the limitation section by indicating the absence of external validation and risk of overfitting and sample imbalance of the participants in terms of residence areas in the revised manuscript. Thank you

# Reviewer report: Reviewer response for version 1

**DOI:** 10.5256/f1000research.171606.r367453

**Published:** 2025-04-02

**Authors:** Christiana Olanrewaju Sowunmi

**Affiliations:** 1 Babcock University, Ilishan-Remo, Ogun State, Nigeria

The study explored an important area constituting reproductive health disease burden especially in sub-Saharan Africa. Given that the contraceptive prevalence (CPR) is low in African countries offers credit to this investigation on identifying factors relating to the problem. Identified major determinants by the authors resonate other studies on MCs’ use not only in Ethiopia but in Nigeria. Utilization of Machine Learning (ML) algorithm in the study is a novel idea and amplifies use of technology – artificial intelligence in health disciplines’ investigations. The six ML algorithms: LR, RF, KNN, XGBoost, NB and SVMs are widely used as study models. Methods of model training and selection were accurate, establishing the XGBoost as the best model for predictors identification, but poor in specificity ability. The rich source of data – Performance Monitoring and Accountability (PMA) Ethiopia 2019 survey dataset provided a large sample for generalization of findings.  Overall, the study adds to the body of knowledge on MCs use, thus preventing abortions from unwanted and unplanned pregnancies. Findings will significantly contribute to the reduction in maternal morbidity and mortality deaths resulting from abortions and related complications.

Is the work clearly and accurately presented and does it cite the current literature?

Yes

If applicable, is the statistical analysis and its interpretation appropriate?

Yes

Are all the source data underlying the results available to ensure full reproducibility?

Yes

Is the study design appropriate and is the work technically sound?

Yes

Are the conclusions drawn adequately supported by the results?

Yes

Are sufficient details of methods and analysis provided to allow replication by others?

Yes

Reviewer Expertise:

Maternal and Child Health

I confirm that I have read this submission and believe that I have an appropriate level of expertise to confirm that it is of an acceptable scientific standard.
